# Doxorubicin combined with celecoxib inhibits tumor growth of medullary thyroid carcinoma in xenografted mice

**DOI:** 10.3892/ol.2014.2050

**Published:** 2014-04-09

**Authors:** XIANYING MENG, QIANG ZHANG, GUIBIN ZHENG, RENZHU PANG, TEBO HUA, SHUAI YANG, JIE LI

**Affiliations:** 1 Department of Thyroid Surgery, First Hospital, Jilin University, Changchun, Jilin 130021, P.R. China; 2 Department of Geratology, First Hospital, Jilin University, Changchun, Jilin 130021, P.R. China

**Keywords:** celecoxib, doxorubicin, medullary thyroid carcinoma, xenograft tumor

## Abstract

The aim of the present study was to investigate the antitumor effect of celecoxib (CXB) combined with doxorubicin (DOX) on the subcutaneous xenograft tumor of medullary thyroid carcinoma in nude mice, and to analyze the possible mechanism of action. Nude mice with xenografted medullary thyroid carcinoma (MTC) were randomly divided into the control, CXB, DOX and DOX plus CXB groups, and the drug treatment was administered for three weeks. It was found that the tumor inhibition rates and the apoptosis index in the treatment groups were higher than in the control group (P<0.01), and that these values were higher in the combination group compared with the single-drug group (P<0.01). DOX alone upregulated the cyclooxygenase-2 and multidrug-resistance 1 expression levels, and the combination of CXB and DOX or CXB alone notably decreased the expression level of the two proteins compared with no treatment. The results of the present study provide evidence that a combination of DOX and CXB is a potential drug candidate for the treatment of MTC.

## Introduction

Primary thyroid cancers account for 1% of all malignant tumors. Although the life expectancy is generally high, these cancers cause more fatalities than all endocrine organ cancers (1). Medullary thyroid carcinomas (MTCs) are the third most common of all the thyroid cancers and are responsible for ~3–4% of all thyroid cancer cases (2). A common discovery in MTCs that are diagnosed late is lymph node metastasis in the upper mediastinum and neck. Metastases occur in ~70% of patients with MTC who have a palpable thyroid nodule (>1.0 cm diameter) (3). At this stage of the disease, patients cannot undergo surgical resection and do not concentrate radioactive iodine (RAI^131^), and biochemical ‘cure’ rates drop to ≤30% (4,5). Therefore, it is important to develop novel therapies for the treatment of MTC.

Doxorubicin (DOX), a broad-spectrum anthracylin, is isolated from *Streptomyces peucetius* and has been used for the treatment of several cancers, including ovarian, breast and prostate cancer (6). Notably, DOX is the most widely used anticancer drug that is approved by the Food and Drug Administration (7). However, studies have shown that specific cancer cells, including those of the thyroid, are resistant to the apoptotic effects of DOX (8). Non-tumorous tissues, including those of the liver, heart and kidney, develop severe side-effects following DOX-based chemotherapy, which limits its clinical applications (9,10). In addition, the severe dose-dependent side-effects, including stomatitis, neurological disturbances, acute nausea and vomiting, myocardial toxicity, alopecia and bone marrow aplasia, also limit the use of DOX (7). Thus, improved therapeutic regimens that potentiate DOX effects, allowing dose reduction and protection of non-tumorous tissues, are required to improve the treatment of patients with MTC.

The mechanisms involved in DOX-mediated cytotoxicity differ between normal tissues and cancer cells (11). DOX toxicity in cancer cells primarily occurs due to its ability to intercalate between the DNA strands to act as a topoisomerase II inhibitor and/or bind covalently to proteins involved in DNA replication and transcription (7). In normal tissue, however, the DOX-induced side-effects, including hepatotoxicity or cardiotoxicity, are mainly due to the generation of oxygen free radicals, which are inhibited by free radical scavengers (12). This difference in DOX-mediated toxicity in cancer and normal cells can be analyzed to improve the antitumor effects of DOX in combination with other antitumor drugs, thus allowing a dose reduction of DOX to protect the normal cells. Combination therapy with DOX has recently gained much attention (13,14). A study by Dayton *et al* (14) found that combining DOX with HO-3867 could reduce myocardial toxicity and enhance cell death through the use of DOX at lower doses. Therefore, combination therapy has been shown to be a productive method of lessening the side-effects associated with DOX, while retaining the therapeutic function of the drug.

Celecoxib (CXB) is a selective cyclooxygenase (COX)-2 inhibitor that has been promoted as an anti-inflammatory drug with improved safety and lower toxicity compared with other non-steroidal anti-inflammatory drugs. Recently, a study has shown that CXB in combination with DOX could increase growth inhibition and apoptosis in acute myeloid leukemia cells compared with treatment of DOX or CXB alone (15). The combination of CXB with DOX may induce significant growth inhibition of neuroblastoma tumors, and prevent and treat neuroblastoma (16). However, the combined effect of DOX and CXB has not been reported in MTC, a mechanism of action has not been determined and a combination treatment has not been tested *in vivo* for the suppression of tumor growth. Thus, the present study aimed to evaluate the effects of the combination of the DOX chemotherapeutic agent and COX on MTC and normal cells. Furthermore, the study examined the effect that a combination treatment *in vivo* had on tumor growth, and the possible mechanism was examined by assessing the expression of multidrug-resistance protein 1 (MDR1) and COX-2 using xenograft tumors produced by injecting thyroid carcinoma TT cells into nude mice.

## Materials and methods

### Reagents

CXB and DOX were obtained from Pfizer, Inc., (New York, NY, USA). Stock solutions of 1 mM CXB (Sigma Aldrich, St. Louis, MO, USA) were dissolved in dimethyl sulfoxide (Sigma Aldrich), stored at −20°C and diluted in fresh medium prior to use. For the western blot analysis, the following antibodies were used: Rabbit monoclonal anti-COX-2 and anti-MDR1 (Cell Signaling Technology, Beverly, MA, USA), mouse monoclonal anti-β-Actin (Sigma Aldrich) and horseradish peroxidase-conjugated goat anti-rabbit immunoglobulin G (IgG; Santa Cruz Biotechnology, Inc., Santa Cruz, CA, USA). All other reagents were obtained from Sigma Aldrich unless otherwise stated.

### Cell culture

The human MTC cell line, TT, was obtained from the cell bank of the Chinese Academy of Sciences (Beijing, China) and the cells were grown in RPMI 1640 medium (Invitrogen, Carlsbad, CA, USA) supplemented with 10% fetal bovine serum (FBS), 100 M non-essential amino acids and 100 mM L-glutamine (Invitrogen) at 37°C in a 5% CO_2_ atmosphere and at 95% humidity.

### Cell viability analysis

The TT cells were incubated at a concentration of 5×10^3^ cells/well in a 96-well plate, and grown at 37°C, with 5% CO_2_ until cell adherence. The cells on the culture plate were divided into groups on the basis of parallel well lines following an overnight incubation in fresh RPMI 1640 containing 0.5% FBS, and each group had four wells in one line. Following the 24-h attachment period, the cells were treated with DOX and CXB either alone or in combination. A total of 20 μl MTT (5 mg/ml) was added and the cells were incubated for another 4 h at the end of the treatment. Subsequent to the removal of the supernatant, 200 μl DMSO was added to each well. The plate was then agitated for 5 min. In the shaking board, cell viability was obtained by measuring the absorbance at 490 nm using an enzyme-labeling instrument (Bio-Tek ELX800; Bio-Tek, Vermont, VT, USA), and this assay was performed in triplicate. The inhibition rate was calculated according to the following formula (17): Inhibition rate (%) = [1 - (average absorbance of experimental group / average absorbance of blank control group)] × 100.

### Apoptosis analysis

The cells were cultured in six-well plates in RPMI 1640 supplemented with 10% FBS medium, and were treated with DOX and CXB alone or with a combination of CXB/DOX for 24, 48 and 72 h. The cover slips were washed three times with phosphate-buffered saline (PBS) and single cell suspensions were fixed in 1% PBS. The cells were stained with 100 μg/ml acridine orange and 100 μg/ml ethidium bromide for 1 min. The cells were then observed under a fluorescence microscope (CKX41-F32FL, Olympus, Tokyo, Japan). At least 200 cells were counted and the percentage of apoptotic cells was determined. Triplicates were performed in all experiments and each experiment was performed three times.

### Human MTC xenograft experiment

For the human MTC xenograft experiment, 4–6-week-old female BALB mice were maintained under specific pathogen-free conditions and provided with food and water *ad libitum*. All the animals were fed with a normal pellet diet one week prior to experimentation. *In vitro* cultured human MTC TT cells were injected subcutaneously into the right supra scapula region. The tumor volume was calculated by the following formula: Volume = (length × width^2^ ) / 2. When tumors grew to an average volume of 75 mm^3^, the mice were randomly divided into four groups (30 mice per group) and treated intragastrically with 300 mg/kg CXB three times a week (CXB group), by intraperitoneal (i.p.) injection with 4 mg/kg DOX once a week (DOX group), by i.p injection of a combination of 2 mg/kg DOX and 150 mg/kg CXB once a week (COX plus DOX group) or injected with the same volume of saline once a week (Control group) for three weeks. The tumor volumes were determined by caliper measurement twice a week. When the control mice started to succumb to their tumors, the mice in all treatment groups were euthanized and the tumors were weighed to determine treatment efficacy. The tumor tissue and liver samples of the mice were isolated for histopathological evaluation. The present animal study was performed following approval of the protocol by the Jilin University Animal Care and Use Committee (Changchun, Jilin, China).

### Quantitative polymerase chain reaction (qPCR) for COX-2 and MDR1 expression

The tumor tissue samples were isolated from sacrificed mice and total RNA was extracted using TRIzol reagent following the manufacturer’s instructions (Invitrogen Life Technologies). RNA was reverse-transcribed into complementary DNA (cDNA) using a Primescript™ RT reagent kit (Takara Bio, Inc., Dalian, China) according to the manufacturer’s instructions. qPCR was performed with the SYBR green fluorescent dye method and a Rotor-Gene 3000 Real-Time PCR machine (Qiagen, Duesseldorf, Germany). COX-2, MDR1 and β-actin primer sequences are listed in Table I. β-actin was used as an internal control to evaluate the relative expressions of COX-2 and MDR1. The PCR conditions were as follows: Pre-denaturing at 95°C for 2 min followed by 45 cycles of denaturation at 95°C for 10 sec and annealing/extension at 59°C for 20 sec. The amplification specificity was checked by melting curve analysis. The PCR products were visualized by gel electrophoresis to confirm the presence of a single product with the correct size. The 2^−ΔΔCT^ method was used to calculate the relative abundance of the target gene expression generated by Rotor-Gene Real-Time Analysis Software 6.1.81 (Qiagen). For each cDNA sample, the target gene mRNA level was normalized to the β-actin mRNA level. The experiments were performed three times.

### Western blot analysis

Protein from the tumor tissue was extracted by the Mammalian Protein Extraction kit (Kangwei Century Co., Ltd., Beijing, China) following the manufacturer’s instructions. The protein concentration was determined by the bicinchoninic acid assay with bovine serum albumin (Sigma Aldrich) as the standard. Western blotting was performed. Briefly, an equal amount of the total cell lysate (50 μg) was solubilized in the sample buffer and boiled for 5 min. A total of 25 μl of this lysate was electrophoresed on an 8% SDS-PAGE gel and then the proteins were transferred to polyvinylidene difluoride membranes (Millipore, Billerica, MA, USA) by transfer buffer at 400 mA for 1 h. Non-specific binding was blocked with 5% skimmed milk powder for 1 h at room temperature. The membranes were incubated with the specific primary antibody overnight at 4°C. The primary antibodies used were the polyclonal rabbit anti-human MDR1 (1:10,000) and rabbit anti-human COX-2 (1:1,000) antibodies. Subsequent to washing three times with Tris-buffered saline plus Tween 20 solution and incubation with the horseradish peroxidase-conjugated goat anti-rabbit IgG as the secondary antibody (1:5000 dilution) for 1 h at room temperature, the bands were visualized with the enhanced chemiluminescence system (GE Healthcare, Little Chalfont, Buckinghamshire, UK). The membranes were then re-blotted with anti-β-actin antibody for normalization and confirmation of equal protein loading.

### Statistical analysis

All the statistical analyses were performed by GraphPad Prism 5.0 software (GraphPad Software, Inc., San Diego, CA, USA). Data are presented as the mean ± standard deviation. The statistical significance was determined using one-way analysis of variance and Student’s t-test. P<0.05 was considered to indicate a statistically significant difference.

## Results

### CXB plus DOX reduces cell viability

**Figure 1:**
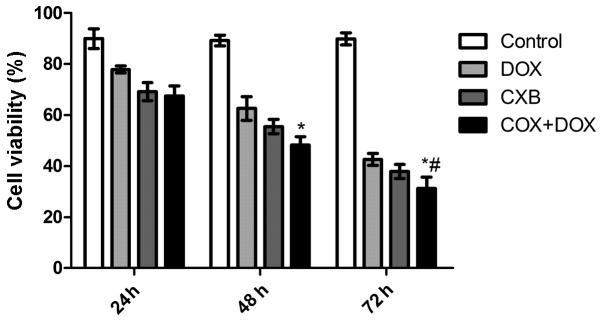
Growth inhibiting effects of CXB, DOX and DOX plus CXB in TT cells. Cell viability was determined by the MTT method. This assay was performed in triplicate. Inhibition of cell growth was observed following 24, 48 and 72 h of treatment (P<0.05, ANOVA). ^*^P<0.05 vs. 24 h; and ^#^P<0.05 vs. CXB. ANOVA, analysis of variance; CXB, celecoxib; DOX, doxorubicin.

To investigate whether DOX combined with CXB inhibits thyroid cancer cell proliferation, TT cells derived from poorly-differentiated human medullary carcinoma cells were treated with CXB, DOX or CXB combined with DOX for 24, 48, and 72 h. The anti-proliferative effect of DOX, CXB and DOX plus CXB on the TT cells was examined by MTT assay. It was found that CXB, DOX or CXB plus DOX could significantly inhibit the proliferation of the TT cells in a time-dependent manner (P=0.011). As shown in Fig. 1, the inhibitory rates of the CXB plus DOX group was higher compared with the COX and DOX groups (P=0.006 and 0.007, respectively). There was no significance difference between the COX and DOX groups (P=0.678).

### CXB plus DOX synergistically induces apoptosis

**Figure 2:**
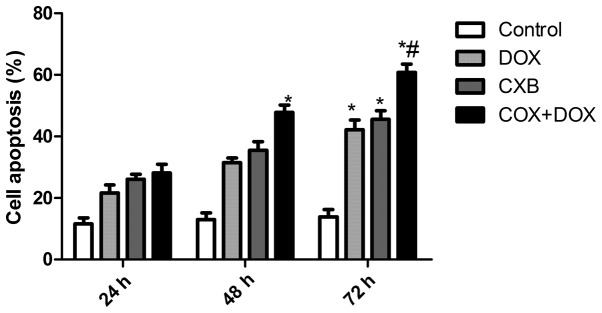
Effect of CXB, DOX and DOX plus CXB on apoptosis of TT cells *in vitro*. This assay was performed in triplicate. The time-dependent apoptosis of the cells was observed following 24, 48, and 72 h of treatment ^*^P<0.05 vs. 24 h; and ^#^P<0.05 vs. CXB. CXB, celecoxib; DOX, doxorubicin.

The effects of DOX and CXB on the cell cycle of the TT cells were analyzed. The TT cells treated with DOX or CXB had an increased percentage of apoptotic cells compared with untreated cells (Fig. 2). The DOX plus CXB combination resulted in an even greater percentage of apoptotic cells compared with the higher doses of either drug alone (P=0.004 and 0.006, respectively). These data are consistent with the results from the MTT assay. Taken together, these results indicate an additive mechanism for DOX and CXB in inducing cell death through apoptosis.

### DOX plus CXB causes significant inhibition of tumor growth

**Figure 3:**
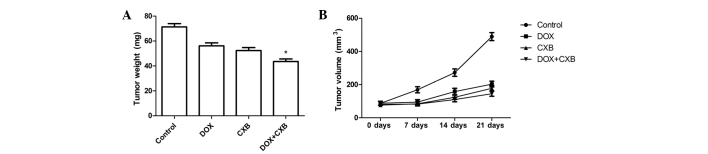
Quantitative analysis of tumor tissue in BALB/c mice following a single or combination treatment of DOX and CXB (^#^P<0.05 vs. control). (A) Tumor weight of untreated and treated mice after 21 days. (B) Tumor volume of treated and untreated mice at days 7, 14 and 21. COX-2, cyclooxygenase-2; DOX, doxorubicin; MDR1, multidrug-resistance protein 1.

The *in vivo* therapeutic efficacy of DOX and CXB was assessed in female BALB mice bearing medullary thyroid tumors. The mice were sacrificed and the tumor tissue was removed 21 days after treatment. The tumor weight of the animals was then measured. It was found that the tumor weight of the treatment group was lower compared with the untreated group. The DOX plus CXB group was lower compared with the single CXB or DOX groups (P=0.02 and 0.03, respectively) (Fig. 3A). There were no significant differences between the COX and DOX groups (P>0.05). Furthermore, the *in vivo* activity of DOX combined with CXB was examined, and it was identified that the growth of the established medullary thyroid tumor xenografts in terms of tumor volume was inhibited when treated with a single or combination of the drugs during days 7, 14 and 21 (Fig. 3B). CXB, DOX and the combination of drugs showed a 45.37, 49.71 and 69.68% decrease in the mean tumor volume at day 21, respectively, compared with tumors from the untreated controls (Fig. 3B). As shown in Fig. 3, DOX combined with CXB resulted in an even greater percentage of tumor inhibition rates compared with either drug alone at the various times (all P<0.05). These results showed that DOX and CXB, but particularly DOX combined with CXB, induced tumor regression and slowed tumor growth in the mice of the treatment groups compared with the untreated group.

### DOX plus CXB effects COX-2 and MDR1 production in MTC tumors

**Figure 4:**
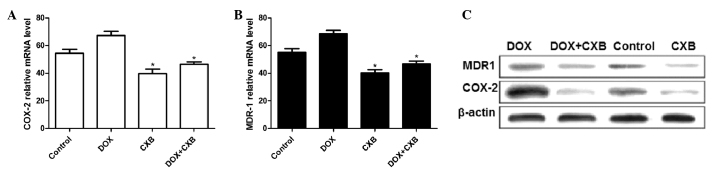
Expression of COX-2 and MDR1 in the tumor tissue was determined following treatment with CXB, DOX or a combination for 21 days. Expression of (A) COX-2 and (B) MDR1 mRNA in tumor tissue, as determined by qPCR. (C) Protein expression level of COX-2 and MDR1 in tumor tissue, as determined by western blot analysis. ^*^P<0.05 vs. DOX. COX-2, cyclooxygenase-2; DOX, doxorubicin; MDR1, multidrugresistance protein 1; qPCR, quantitative polymerase chain reaction.

The COX-2 and MDR1 mRNA and protein expression levels were examined by qPCR and western blot analysis, respectively. The COX-2 and MDR1 expression at the mRNA level decreased following treatment with CXB or the combination of DOX and CXB compared with the untreated and DOX groups (all P<0.05) (Fig. 4A and B). Additionally, COX-2 and MDR1 expression of the mRNA in the DOX group was higher compared with the untreated group. DOX alone considerably upregulated the MDR1 and COX-2 protein expression level and the combination of CXB and DOX, or CXB alone notably decreased the protein expression levels compared with no treatment (Fig. 4C).

## Discussion

The resistance of MTC to conventional chemotherapy drugs is the major reason for the high mortality rate of patients with MTC who fail to respond to surgery. Novel target therapies have been under evaluation for the treatment of advanced MTC cases in the last 10 years. Although certain promising antitumor drugs have been used in phase II/III clinical trials previously (18–21), certain patients cannot be enrolled due to the extremely restrictive inclusion criteria or they drop out of the trial due to severe side-effects. DOX is the most widely used anticancer drug, and despite being previously used for the treatment of several cancers, including ovarian, breast and prostate cancer (6), its clinical use is limited by its toxicity to normal tissues, such as those of the heart and liver. Another limitation of its effectiveness is the development of multidrug resistance by cancer cells. Thus, combination therapy has proven to be a useful method in reducing the side-effects associated with DOX for the treatment of patients with MTC.

A previous study has shown that the combination of DOX with rofecoxib, as a selective COX-2 inhibitor, could reduce TT cell growth (22). This is in agreement with the results of the present study, which showed that DOX plus CXB could reduce cell viability with a significant increase in apoptosis compared with a single-drug treatment. Additionally, a study by Vivaldi *et al* (23) identified that a combination of CXB and vinorelbine, but not DOX, induced a significant reduction in cell viability and an increase in apoptosis *in vitro*, which is also in agreement with the results of the present study. These studies and the results of the present study further show that combination therapy may be a useful method in treating patients with cancer.

DOX treatment has led to partial biochemical and tumor responses in a few patients, ranging from 10–20% of treated cases (24), which showed that the chemotherapy for metastatic MTC has a limited efficacy. Multidrug resistance is one of the mechanisms for the resistance of MTC to conventional chemotherapy (25). MDR in cancer cells has been attributed to the overexpression of several plasma membrane adenosine triphosphate (ATP)-dependent efflux pumps, including P-glycoprotein (P-gp), which is encoded by the ATP-binding cassette, sub-family B, member 1 gene, also named MDR1 (26), breast cancer-resistance protein (BCRP), which is encoded by the BCRP gene (27) or multidrug-resistance proteins (MRP1–3), which are encoded by the MRP genes (28). A previous study has shown that the COX-2 gene is able to regulate MDR1 expression in rat mesangial cells (29), and that the use of a COX-2 inhibitor, rofecoxib, was able to sensitize MTC cells to DOX in the treatment of an MTC-derived cell line *in vitro* (22). It has been found that COX-2 expression is able to induce the expression of the MDR1 gene, which codes for the P-gp efflux pump, which pumps numerous drugs out of cells (29). This association indicates that the inhibition of COX-2 by specific inhibitors, including CXB, rofecoxib or others, may improve the sensitivity of cancer cells to chemotherapy. In the present study, the data confirmed that CXB, a COX-2 inhibitor, could improve the sensitivity of the MTC cells to DOX and decrease the effective dose of DOX that must be used.

In the present study, the effect of CXB on the mRNA and protein expression of MDR1 and COX-2 was examined by qPCR and immunohistochemistry, respectively. It was found that CXB alone or in combination with DOX could decrease MDR1 and COX-2 expression, which complies with previous results (23). As MDR1 codes for the P-gp efflux pump, it is accepted that the action of CXB on drug efflux is exerted through the inhibition of MDR1 expression. In addition, the present results showed that DOX alone was able to increase MDR1 and COX-2 expression, which showed that DOX has multidrug resistance for metastatic MTC, and therefore, treatment of metastatic MTC by a combination of DOX with COX-2 inhibitors is an effective method. Despite the anti-tumor activity of COX-2 inhibitors, particularly CXB, they are effective against a wide variety of human epithelial tumor types, including colorectal, non-small cell lung, breast and prostate cancers (30). To the best of our knowledge, the present study is the first to show that DOX plus CXB is able to decrease tumor growth *in vitro* and *in vivo.*

In conclusion, in the present study it has been shown that CXB, a COX-2 inhibitor, in combination with DOX, a chemotherapeutic drug, is able to induce MTC cell apoptosis and reduce tumor growth *in vivo*. Furthermore, CXB could enhance the chemotherapeutic effect of this drug by the inhibition of COX-2 and MDR1 expression.

## Figures and Tables

**Table I:** PCR primers for the objective gene.

Gene	Primer sequences (5′-3′)	Fragment
MDR1		
Forward	ACCGCAAACGCTTTATGCTG	158
Reverse	ACGAGCTATGGCAATGCGTT	
COX-2		
Forward	ACCGCAAACGCTTTATGCTG	179
Reverse	AAAGATGGCATCTGGCGGA	
β-actin		
Forward	GTTGCGTTACACCCTTTCTTG	142
Reverse	TGCTGTCACCTTCACCGTTC	

PCR, polymerase chain reaction; MDR1, multidrug-resistance protein 1; COX-2, cyclooxygenase-2.
